# The impact of endometrioma and laparoscopic cystectomy on ovarian reserve and the exploration of related factors assessed by serum anti-Mullerian hormone: a prospective cohort study

**DOI:** 10.1186/s13048-014-0108-0

**Published:** 2014-11-26

**Authors:** Yuqing Chen, Huihui Pei, Yajie Chang, Minghui Chen, Haihe Wang, Hongzhe Xie, Shuzhong Yao

**Affiliations:** The First Affiliated Hospital, Sun Yat-sen University, Guangzhou, China; The People’s Hospital of Anyang City, Anyang, China; The Sixth Affiliated Hospital, Sun Yat-sen University, Guangzhou, China

**Keywords:** Anti-Müllerian hormone, Endometrioma, Ovarian cystectomy

## Abstract

**Background:**

To evaluate the impact of the presence of endometrioma and laparoscopic cystectomy on ovarian reserve as assessed by serum anti-Müllerian hormone (AMH) level. In addition, factors related to the decline in ovarian reserve were analyzed.

**Methods:**

From June 2013 to January 2014, we prospectively included 40 women with endometriomas as the study group (group A), 36 women with tubal factor infertilities as control group 1 (group B) and 22 women with the other benign ovarian cysts as control group 2 (group C). The women with ovarian cysts underwent laparoscopic cystectomy. Serum AMH levels were determined preoperatively and at 1 month after surgery.

**Results:**

The endometrioma group had lower AMH levels (1.53 ± 1.37 ng/ml) compared with the other benign ovarian cyst group (2.20 ± 1.23 ng/ml) and the tubal factor infertility group (2.82 ± 1.74 ng/ml). The rate of serum AMH decline 1 month after surgery in the endometrioma group (0.62 ± 0.35) was larger than the decline in the other benign ovarian cyst group (0.32 ± 0.30). The preoperative AMH level showed a significant correlation with patient age (group A, r = −0.32; group B, r = −0.54; group C, r = −0.71); there was a statistically significant correlation between the rate of serum AMH decline and endometrioma diameter as well as with the preoperative serum AMH level. In addition, the rate of serum AMH decline was larger for bilateral endometriomas than for unilateral endometriomas, but there was no similar correlation in the other benign ovarian cyst group. The rate of AMH decline after surgery in the subgroup of >7 cm was significantly higher than in the subgroup of ≤7 cm.

**Conclusions:**

Ovarian endometriomas per se may damage ovarian reserve, and cystectomy of endometriomas may cause greater damage to ovarian reserve compared with other benign ovarian cysts. The operation-related damage to the ovarian reserve was positively related to whether the endometriomas were bilateral, as well as cyst size (especially for cysts >7 cm), but was negatively related to the preoperative serum AMH level. Age was a negative factor that affected the ovarian reserve.

## Background

Endometriosis is an enigmatic disease characterized by the development of the endometrial tissue outside of the uterus. The most common location of endometriosis is the ovary, occurring in 17-44% of patients affected by endometriosis [1]. Laparoscopic excision of endometriomas is regarded as a first-line treatment, but the reduced number of retrieved oocytes for in vitro fertilization and premature ovarian failure after surgery reported in several papers have raised concerns [2,3]. Because endometriosis is most common in reproductive-age women, it is important to predict and protect the patients’ ovarian function. Ovarian reserve is defined as the functional potential of the ovary, and it reflects the number and quality of the follicles left in the ovary at any given time. At present, the ideal test reflecting ovarian reserve remains the serum anti-Müllerian hormone (AMH) level, which has equal sensitivity and specificity to the antral follicle count (AFC) and is better than follicle-stimulating hormone (FSH), estradiol, luteinizing hormone (LH), FSH/LH ratio or inhibin-B levels [4]. Moreover, the serum AMH level appears to be independent of the menstrual cycle and is not affected by the use of gonadotropin-releasing hormone (GnRH) agonists or oral contraceptives [5-8]. It is worthwhile and convenient to elucidate changes in ovarian reserve as measured by serum AMH level, which reflects the count of primordial follicles, after ovarian surgery.

To date, there have been several studies of the effect of laparoscopic cystectomy on ovarian reserve, but controversies still exist. There are no definitive data to clarify whether the damage to the ovarian reserve observed in patients with endometriomas is related to the surgical procedure, to the previous presence of a cyst, or both. It is also unclear what the risk factors for damage to the ovarian reserve after surgery are.

Thus, our prospective cohort study analyzed the preoperative serum AMH level and changes in the serum AMH level after surgery in the endometrioma group by comparing these patients with tubal factor infertility patients and patients with other benign ovary cysts to identify differences in the preoperative serum AMH levels, changes after surgery and the determinants of surgery-related changes on ovarian reserve.

## Methods

### Patients

The present prospective cohort study recruited a total of 98 women who underwent laparoscopic surgery due to endometriomas (n = 40), other benign ovarian cysts (n = 22; 18 teratomas, 2 mucinous cystadenomas and 2 serous cystadenomas) and tubal factor infertility (n = 36) from June 2013 to January 2014 at the Department of Obstetrics and Gynecology of the First Affiliated Hospital of Sun Yat-sen University in Guangzhou, China. All the patients with endometriomas or other benign ovarian cysts were confirmed by pathology. And the routine approach for tubal factor infertility was as follows: 1. history; 2. ultrasound; 3. sex hormone and ovulation supervision; 4. sperm analysis; and 5. hysteroscopy, laparoscopy and hysterosalpingography. The general clinical data, surgical data and sera were collected preoperatively and 1 month after surgery. This study was approved by the Institutional Review Board of the First Affiliated Hospital of Sun Yat-sen University, and informed consent was obtained from all of the patients.

For the endometrioma group (study group, group A) and the other benign ovary cyst group (control 2, group C), the inclusion criteria were as follows: (1) previous diagnostic procedures for ovarian tumors and histologically confirmed endometriomas or other benign ovarian cysts; (2) 20- to 40-year-old women with regular menstrual cycles (21–35 days) at the time of the operation; and (3) no evidence of any endocrine disorders, such as polycystic ovarian syndrome (PCOS), diabetes mellitus, thyroid dysfunction, hyperprolactinemia, congenital adrenal hyperplasia, Cushing’s syndrome, systemic lupus erythematosus (SLE) or adrenal insufficiency. The exclusion criteria were as follows: (1) postmenopausal status at the time of the operation; (2) a history of adnexal surgery; (3) previous history of adnexal surgery; (4) any suspicious findings of malignant ovarian diseases; and (5) any hormone treatment within 3 months prior to enrollment.

Our indications for surgery in the endometrioma group were as follows: 1. infertility; 2. chronic pelvic pain; 3. cyst size >5 cm lasting for more than two menstrual periods; and 4.malignancy cannot be excluded. If the patients were not infertile, then a cyst size >5 cm was the threshold. If the patients were infertile, then cystectomy was performed regardless of cyst size.

For the tubal factor infertility group (control 1, group B), inclusion was based on infertility caused by the fallopian tube (based on: 1. history; 2. ultrasound; 3. sex hormone and ovulation supervision; 4. sperm analysis; and 5. hysteroscopy, laparoscopy and hysterosalpingography.), and cases were excluded if a salpingectomy was performed during the surgery or if there was any evidence of ovarian cysts or endometriosis. All of the other inclusion and exclusion criteria were the same as noted above.

### Surgery

All of the women underwent the operation under general anesthesia. Laparoscopic pneumoperitoneum was induced by CO_2_ insufflation using a Veress needle that was passed through a 1-cm umbilical incision until the intra-abdominal pressure reached 12 mmHg. Following pneumoperitoneum, umbilical 10-mm trocar and telescope entries were made. Then, two trocars (5–10 mm) were inserted into both suprainguinal regions under direct laparoscopic observation.

After adhesiolysis and mobilization of the ovary, we determined whether the ovarian cyst was an endometrioma. Then, the following steps were performed: incision of the ovarian cyst with monopolar scissors, aspiration of the contents of the cyst, and stripping of the cyst using two atraumatic grasping forceps by traction and countertraction after the identification of the cleavage plane.

If the cyst was another type of benign cyst, then the normal ovarian cortical tissues on the cysts were incised to the level of the cyst capsule, and the cyst was removed from the ovarian cortex by traction with a grasping forceps. Every effort was made to remove the cyst without spilling the contents.

Next, sutures were used to reapproximate the ovarian edges and obtain hemostatic control. When necessary, hemostasis was achieved with bipolar forceps applied to the ovarian parenchyma. To reduce the possibility of damage to the normal ovarian tissue, hemostatic procedures were minimized. Following the removal of the ovarian cyst, the specimens were assessed by visual examination for any evidence of malignancy. Histological classification of the specimens was then performed.

For the tubal infertility group, laparoscopic adhesiolysis, hydrotubation and hysteroscopy were performed.

All of the laparoscopic procedures were performed by the same skilled surgical team.

### Hormonal measurements

Serum samples from each patient were collected preoperatively and at 1 month after the surgery. The patients’ sera were obtained from blood samples centrifuged at 3000 rpm for 10 minutes to separate the cellular contents and debris. The serum was transferred to sterile polypropylene tubes and stored at -80C until assayed.

The serum AMH concentration was measured using an enzyme-linked immunosorbent assay (ELISA) kit according to the manufacturer’s instructions (AMH ELISA; Ansh Labs, UK). The minimum detectable concentration for AMH was 0.06 ng/mL. Intra-assay coefficient of variation was 0.02 (2.22/95); Inter-assay coefficient of variation was 7.81 (15.62/2).

### Statistical analysis

Categorical variables were presented as percentages and were compared using the chi-squared test. Continuous variables were presented as $\overline{X}\pm S$. After the Kolmogorov-Smirnov test, the preoperative serum AMH levels were compared using the Kruskal-Wallis test, and changes in the serum AMH levels after surgery were compared within groups using the Wilcoxon matched-pairs signed ranks sum test and between groups using the Mann–Whitney *U* test.

Correlations between several factors and preoperative serum AMH levels and the rate of decline of the AMH levels in the endometrioma group, the infertility group and the other benign ovarian cyst group were analyzed by bivariate correlation analysis and were expressed as Spearman correlation coefficients. Statistical analyses were performed using Statistical Package for the Social Sciences (SPSS, version 13.0). P < 0.05 was considered statistically significant.

## Results

The clinical characteristics, preoperative AMH levels, postoperative AMH levels, and rate of decline of the AMH levels in the endometrioma group, the infertility group and the other benign ovarian cyst group were summarized in Table 1. The differences in age, body mass index (BMI), gravidity, cyst size, and unilateral/bilateral cysts were not statistically significant among the three groups, while the differences in the operating time and intraoperative blood loss were statistically significant (both P < 0.001); a further comparison suggested that the operating time for group B was the shortest (P < 0.001 and P = 0.02 compared with group A and group C, respectively), whereas the difference between groups A and C was statistically insignificant (P = 0.08). The intraoperative blood loss was greatest in group A (P < 0.001 compared to both groups), whereas the difference between groups B and C was statistically insignificant (P = 0.89).

**Table 1:** **Clinical characteristics and serum AMH changes in the three groups**

	**Endometrioma group (n = 40)**	**Tubal factor infertility group (n = 36)**	**Other benign ovarian cyst group (n = 22)**	***P***
Age (y)	30.38 ± 5.13	30.53 ± 4.55	29.95 ± 3.92	0.89
BMI(kg/m^2^)	20.44 ± 2.55	21.19 ± 2.58	20.19 ± 2.58	0.31
Gravidity	0.90 ± 0.18	1.00 ± 0.22	0.91 ± 0.35	0.64
Cyst size (cm)	7.70 ± 3.66		6.35 ± 2.88	0.15
Bilaterality (%)	16 (40)		4 (18)	0.08
Duration of operative procedure (min)	88.38 ± 45.27	50.50 ± 31.84	67.95 ± 32.32	<0.001
Blood loss (ml)	65.00 ± 44.89	30.56 ± 21.80	28.64 ± 13.20	<0.001
serum AMH (ng/ml)				
Preoperative	1.53 ± 1.37^a^	2.82 ± 1.74^c^	2.20 ± 1.23^b^	*0.001*
Postoperative	0.69 ± 0.89^a^	2.80 ± 1.57^c^	1.48 ± 0.86^b^	
Rate of decline	0.62 ± 0.35	0.02 ± 0.15	0.32 ± 0.30	*<0.001*

Note: 1). The cyst diameter refers to the average value of the maximum diameter line over two perpendicular planes of the ovarian cyst when the lesion is unilateral and refers to the sum of the diameters of the two cysts on both sides when the lesions are bilateral. 2). AMH decline rate = (preoperative AMH level-postoperative AMH level)/preoperative AMH level. 3). For the paired rank-sum test of ^a^ and ^b^, both *P* < 0.001. 4). For the paired rank-sum test of ^c^, *P* = 0.48.

The difference in preoperative serum AMH level among the three groups was statistically significant (P = 0.001); a further comparison indicated that the difference between groups B and C was statistically insignificant (P = 0.29), while the differences between groups A and B and between groups A and C were statistically significant (P < 0.001 and P = 0.02, respectively), indicating that the preoperative serum AMH level in group A was lower than in the other two groups.

Serum AMH levels were decreased at 1 month after laparoscopic ovarian cystectomy in both groups A and C (both P < 0.001), but not in group B (P = 0.48). The rate of serum AMH level decline after surgery in the three groups was significantly different (P < 0.001); a further comparison indicated that the rate of serum AMH level decline in group A was higher than in group C (P = 0.001), and both were higher than in group B (both P < 0.001).

Several univariate analyses were performed to identify factors that were statistically significantly correlated or associated with the preoperative serum AMH and the rate of decline of serum AMH levels (Table 2).

**Table 2:** **Factors correlated with preoperative serum AMH level and the rate of decline level after surgery**

	**Preoperative serum AMH level**						**The rate of decline of the AMH level**			
	**Endometrioma group**		**Tubal factor infertility group**		**The other benign ovarian cyst group**		**Endometrioma group**		**The other benign ovarian cyst group**	
	*P*	r	*P*	r	*P*	r	*P*	r	*P*	r
Age	0.04	−0.32	0.001	−0.54	0.001	−0.71	0.09		0.84	
BMI	0.67		0.25		0.16		0.98		0.37	
Cyst size	0.99				0.55		0.01	0.40	0.17	
Preoperative serum AMH							0.02	−0.37	0.88	
	Preoperative serum AMH	*P*			Preoperative serum AMH	*P*	Serum AMH decline rate	*P*	Serum AMH decline rate	*P*
Unilateral	1.56 ± 1.17^a^	0.52			2.28 ± 1.25^b^	0.47	0.46 ± 0.34^c^	<0.001	0.32 ± 0.23^d^	0.60
Bilateral	1.49 ± 1.66^a^				1.66 ± 1.24^b^		0.85 ± 0.19^c^		0.33 ± 0.66^d^	

Note: ^a,b,c,d^Derived from the Mann–Whitney *U* test for two independent samples.

**Figure 1:**
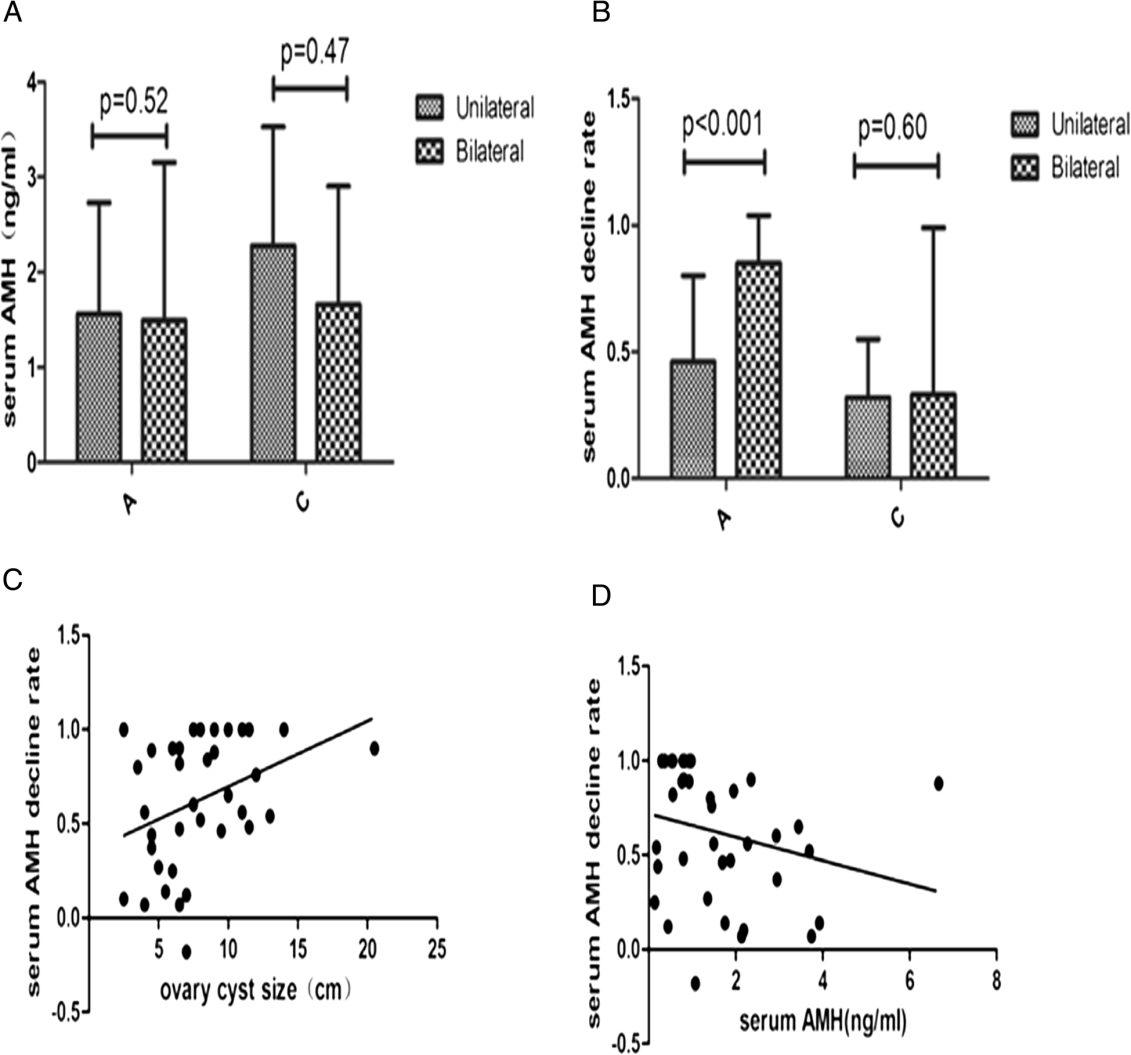
**Factors correlated with preoperative serum AMH level and the rate of decline level after surgery. A** and **B**: Comparison of preoperative serum AMH level and rate of serum AMH level decline between patients with unilateral and bilateral cysts in groups **A** and **C**. **C** and **D**: Scatter plot of the correlation between the rate of serum AMH level decline, cyst size and the preoperative AMH level in the group **A**. The preoperative AMH level showed no difference between unilateral and bilateral cysts in the two groups (Figure 1A). The rate of serum AMH level decline for bilateral cysts was larger than for unilateral cyst in the endometrioma group but not the other benign ovarian cyst group (Figure 1B). The preoperative serum AMH level and cyst size correlated with the rate of serum AMH level decline in the endometrioma group (Figures 1C and 1D).

The preoperative AMH levels showed a significant correlation with patient age (group A, r = −0.32; group B, r = −0.54; group C, r = −0.71) but did not exhibit a significant correlation with BMI, the diameter of the ovarian cyst or the presence of unilateral/bilateral cysts in each group (Figure 1A).

We did not observe any statistically significant correlation between age or BMI and the rate of decline of the AMH levels in the endometrioma group or the other benign ovarian cyst group. However, the preoperative serum AMH level and cyst size correlated with the rate of serum AMH level decline in the endometrioma group (Figure 1C and 1D) but not the other benign ovarian cyst group. In addition, the rate of serum AMH level decline for bilateral cysts was larger than for unilateral cysts in the endometrioma group but not in the other benign ovarian cyst group (Figure 1B).

**Figure 2:**
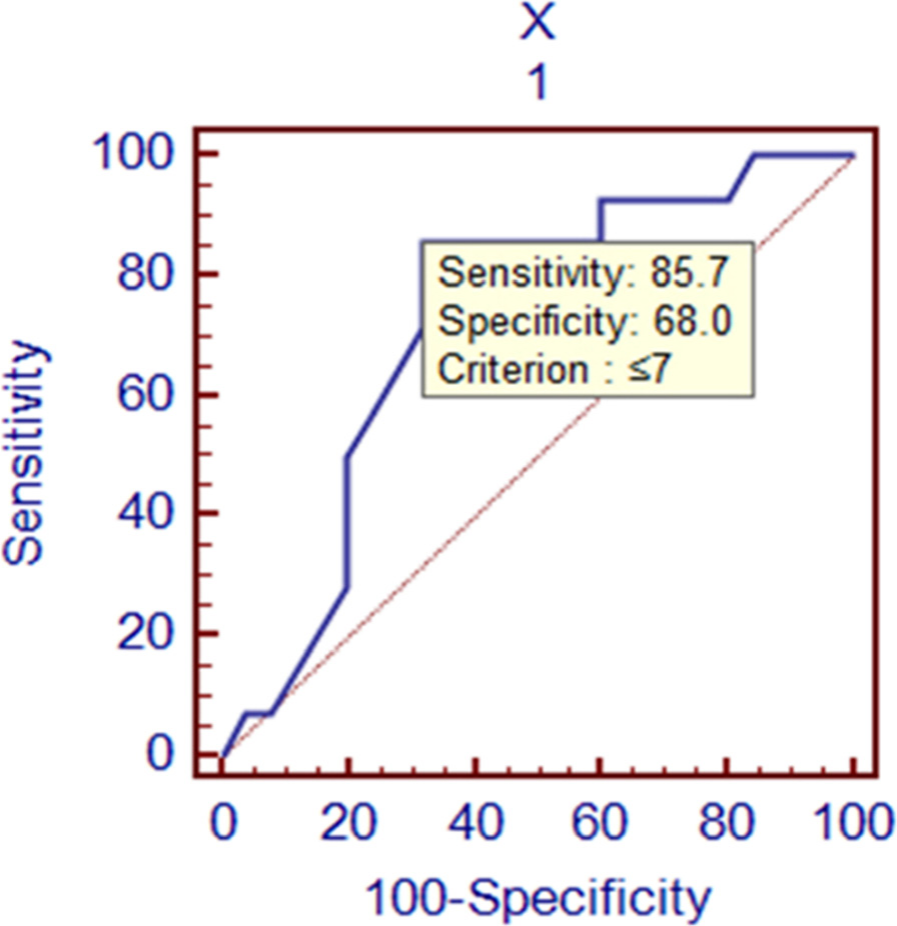
**ROC for rate of serum AMH level decline and endometrioma size.** The area under the ROC curve (AUC) was 0.72 (P = 0.01), indicating that the sensitivity of the serum AMH level declining by 50% or less after cystectomy was 85.7% for a patient with an endometrioma ≤7 cm, and the corresponding specificity was 68.0%.

Because the rate of serum AMH level decline was correlated with cyst size in the endometrioma group, the receiver operating characteristic (ROC) curve was then determined using a serum AMH decline rate of 50% or lower as a cut-off point. As shown in Figure 2, the area under the ROC curve (AUC) was 0.72 (P = 0.01), indicating that the sensitivity of a serum AMH level decline of 50% or less after cystectomy was 85.7% for a patient with an endometrioma ≤7 cm, and the corresponding specificity was 68.0%. Additionally, the rate of decline of the AMH level after surgery in the subgroup with cysts >7 cm was significantly higher than in the subgroup with cysts ≤7 cm (P = 0.001).

## Discussion

### Impact of endometriomas on ovarian reserve and relevant factors

Endometrioma is a common gynecological disease. At present, studies focusing on the impact of endometriomas on ovarian reserve are controversial [9-12]. Therefore, we conducted this study using patients with endometriomas as the study group and patients with other benign ovarian cysts or tubal factor infertility as controls. Our results showed that the preoperative serum AMH levels in patients with endometriomas were significantly lower than in patients with tubal factor infertility or other benign ovarian cysts, and there was no significant difference between patients with other benign ovarian cysts and patients with tubal factor infertility. These results indicate that, in contrast to other benign ovarian cysts, endometriomas per se might damage the ovarian reserve. This finding could be explained based on previous findings. For example, Muzii [13] reported that the ovarian tissue adjacent to an endometrioma was often destroyed and lost its typical follicular structure, while the ovary tissue around other benign ovarian cysts did not exhibit these changes. Stilley [14] stated that peritoneal macrophages in patients with endometriosis were dysfunctional and could secrete proteinases, damaging the ovarian tissue and ultimately leading to a decline in the ovarian reserve.

The analysis of the factors that affect the preoperative serum AMH level showed that neither ovary cyst size nor BMI was related to the preoperative serum AMH level. There was also no relationship with the presence of unilateral or bilateral ovarian cysts. However, for the patients in all three groups, the preoperative serum AMH was negatively correlated with age, indicating that age was the main factor that affected the basal ovarian reserve, which is consistent with recent studies [15].

### Impact of cystectomy on the ovarian reserve and the analysis of relevant factors

Cystectomy is currently the first option for treating ovarian endometriomas [16], but it remains controversial whether the surgery itself decreases the ovarian reserve [17-23]. In this study, the results indicated that for both the patients with endometriomas and patients with other benign ovarian cysts, the serum AMH levels 1 month after cystectomy were statistically significantly lower than the preoperative levels, indicating that cystectomy might do harm to the ovarian reserve. In addition, the serum AMH level declined more in the endometrioma patients than in patients with other benign ovarian cysts, implying that the greater damage to the ovarian reserve caused by cystectomy of endometriomas compared with other benign ovarian cysts might be attributed to the intrinsic characteristics of endometriomas themselves. The pathogenic mechanisms were as follows: 1) inversion and progressive invagination of the ovarian cortex after the accumulation of menstrual debris deriving from bleeding of superficial endometriotic implants [24] and 2) secondary involvement of functional ovarian cysts by endometrial implants located on the ovarian surface [25]. Both theories suggest that the boundary between the endometrial cyst wall and the surrounding normal ovarian tissue is unclear, unlike the boundary in other benign ovarian cysts; thus, removal may cause greater damage to the ovarian reserve. Moreover, the intraoperative blood loss was greater and the operative time was longer in the endometrioma group than in the other benign ovarian cyst group, indirectly indicating that the boundaries between the endometrial cyst and the surrounding ovarian tissue are unclear and that the surgery is more difficult, which supports the suggestion that the surgical removal of endometriomas leads to a higher risk of damaging the ovarian reserve.

Although cystectomy of endometriomas is more likely to damage the ovarian reserve, it is still the first option for treating an endometrioma. One reason is that we cannot completely exclude the possibility of malignant ovarian masses because preoperative pathological examinations are not usually conducted. Moreover, previous studies [26] have confirmed that the risk of ovarian cancer in patients with endometrioma is double the risk in the normal population, and the risk increases to four times when accompanied by infertility. In addition, during the in vitro fertilization/embryo transfer (IVF/ET) process for infertile patients with endometriomas, there is the risk of unintentionally rupturing the endometrioma during oocyte retrieval, which could cause contamination and infection [27].

To reduce the damage to the ovarian reserve caused by cystectomy, in addition to improving surgical skills, we should also pay attention to the relevant factors that affect the damage. In this study, the size of the endometrioma and the presence of bilateral endometriomas were both positively related to the rate of decline of the serum AMH level, whereas this correlation was not observed in patients with other benign ovarian cysts. This result suggested that the removal of bilateral or relatively large endometriomas would cause relatively significant damage to the ovarian reserve. Because of the unclear boundaries between the endometrioma and surrounding ovarian tissue, when the endometrioma is large or is bilateral, the contact area with normal ovarian tissue is large. Consequently, during the surgical removal of the endometrial cyst, it is more likely that a greater amount of the surrounding normal ovarian tissue or the cortex will be removed, causing greater damage to the ovarian reserve. Additionally, further analysis indicated that the serum AMH level after cystectomy of a unilateral endometrioma with a diameter ≤7 cm or bilateral endometrial cysts with total diameters ≤7 cm declined less than for measurements >7 cm. All of these results suggested that ovarian endometriomas should be diagnosed and treated as early as possible, which supports the suggestion of Kitajima *et al.* [28]*.* Moreover, the results also indicated that age was negatively related to the preoperative serum AMH level, although it was not correlated with the rate of decline of the serum AMH level after endometrioma cystectomy. The preoperative serum AMH level itself was negatively related to the decline of serum AMH level following surgery. Therefore, this result implied that it was necessary to measure the preoperative ovarian reserve prior to cystectomy in older patients. If the preoperative serum AMH is too low, then we should consider evaluating the risk of postoperative ovarian failure.

## Conclusions

In summary, ovarian endometrioma per se may damage the ovarian reserve, and compared with other benign ovarian cysts, cystectomy of endometriomas can cause greater damage to the ovarian reserve. This finding supports the idea that ‘endometriosis itself may cause the decline in serum AMH levels, and surgery contributes to this decline by harming healthy ovarian cortex’. The surgery-related damage to the ovarian reserve is positively related to whether the endometriomas are bilateral and to the cyst size and is negatively related to the preoperative serum AMH level of the patients. Age itself is a negative factor that affects the ovarian reserve. Therefore, the treatment plan for patients with ovarian endometrioma should combine individual factors, such as the child-bearing demand, age, characteristics of the cyst and baseline serum AMH level; furthermore, gynecological and reproductive endocrine doctors should work together to develop more rational treatment programs and to provide more guidance for reproductive health care.
